# Delayed presentation of amiodarone extravasation-induced skin necrosis: A case report with discussion on management

**DOI:** 10.1016/j.jdcr.2025.06.006

**Published:** 2025-06-18

**Authors:** Rojoana Rojony, Syeda Beenish Bareeqa, Shivam Singla

**Affiliations:** Department of Internal Medicine, Tidalhealth Peninsula Regional Medical Center, Salisbury, Maryland

**Keywords:** amiodarone, extravasation, skin necrosis, hyaluronidase

**Learning Objectives**

• Early clinical diagnosis after extravasation is crucial in limiting the damage.

## Introduction

Amiodarone is an antiarrhythmic medication, commonly used for treating supraventricular and ventricular arrhythmias.^1^ In an acute setting, especially in unstable patients, the intravenous (IV) route of Amiodarone administration is preferred; however, it is not risk-free. One of the lesser-known but potentially serious complications of the intravenous route is extravasation, where the drug leaks into surrounding tissue. While extravasation injuries are more frequently associated with long-term infusion therapies such as chemotherapy or hyperosmolar agents, amiodarone has also been linked to significant soft tissue damage, including skin necrosis.

The precise mechanism of amiodarone-induced skin necrosis is not fully understood, but it is thought to result from a combination of direct toxicity from excipients like polysorbate 80 and benzyl alcohol, as well as highly acidic pH. It results in local ischemia, causing endothelial damage and increased vascular permeability.^2^ Clinically, patients might experience pain, redness, and swelling at the site of infusion, which can progress to blistering, ulceration, and even full-thickness necrosis, sometimes requiring surgical intervention.^3^ Given the potential severity of this complication, early recognition and quick management are important to prevent long-term damage.

This case report presents a rare but serious complication of amiodarone extravasation leading to skin necrosis, shedding light on clinical presentation and management.

## Case presentation

This case report has been prepared in compliance with the Case Report guidelines to ensure transparency and standardization.^4^

A 30-year-old male with a history of Lennox-Gastaut syndrome, developmental delay (nonverbal at baseline), mild intermittent asthma, stage 3a chronic kidney disease, presented to the emergency department with a 1-day history of fever and shortness of breath.

On admission, he was tachycardic, febrile, and tachypneic. The physical exam was positive for rhonchi, and he was diagnosed with sepsis due to pneumonia, after which antibiotics were started. Two days later, he developed atrial fibrillation rhythm with rapid ventricular response and underwent pharmacological cardioversion with intravenous amiodarone via a large-bore peripheral IV placed in the antecubital fossa cannula. The amiodarone was diluted in dextrose at a concentration of 150 mg/100 mL (1.5 mg/mL) for the loading dose and was administered once at 600 mL/hr over 10 minutes using a 0.22-micron inline filter. Unfortunately, 10 minutes later, the cannula became tissued, leading to significant extravasation of amiodarone into the soft tissue. The affected arm was elevated, and warm compresses were applied by nursing staff. The patient developed mild swelling in the elbow and forearm, but no clinical signs of significant tissue damage were evident on examination. Therefore, subcutaneous hyaluronidase administration was deferred. On the day of extravasation, the patient also developed aspiration pneumonia, became hypoxic, was intubated, and subsequently transferred to the intensive care unit. Due to being intubated, sedated, and nonverbal, the patient was unable to express pain at the site of extravasation.

**Fig 1:**
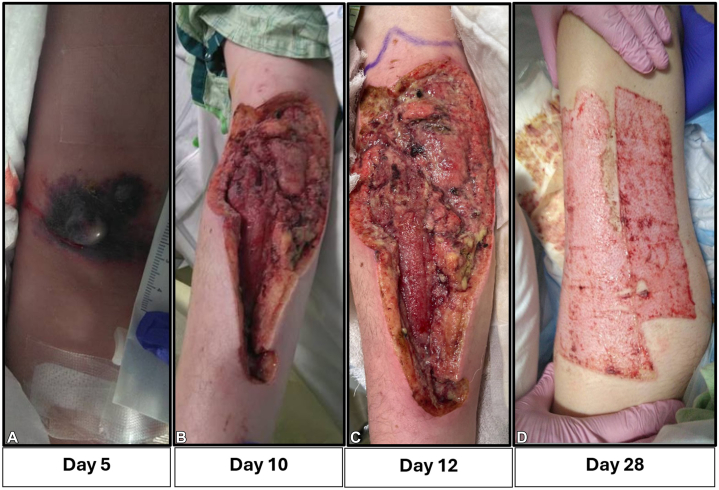
The toxic effects of the amiodarone infusion with sequential changes following I&D and skin grafting. **A,** Day 5 after extravasation, his skin showed bruising, ecchymosis, swelling, and blistering on the flexor aspect of the right forearm and lower arm. **B,** Day 10, after abscess I&D, large pustule excision, and extensive necrotic tissue debridement for 2 times. tissue was removed, which included both skin, subcutaneous tissue, fascia, and parts of the muscle. Marked erythema near right antecubital fossa, exposed muscle is slightly discolored, but all is viable, no necrotic tissue. A small amount of drainage is present. **C,** Day 12, Wound is healing well. **D,** Day 28 split-thickness skin grafting to his right forearm with left upper thigh donor site. Surgical sites have been healing well. *I&D*, Incision and debridement.

A Detailed timeline is provided in Fig 1.

**Fig 2:**
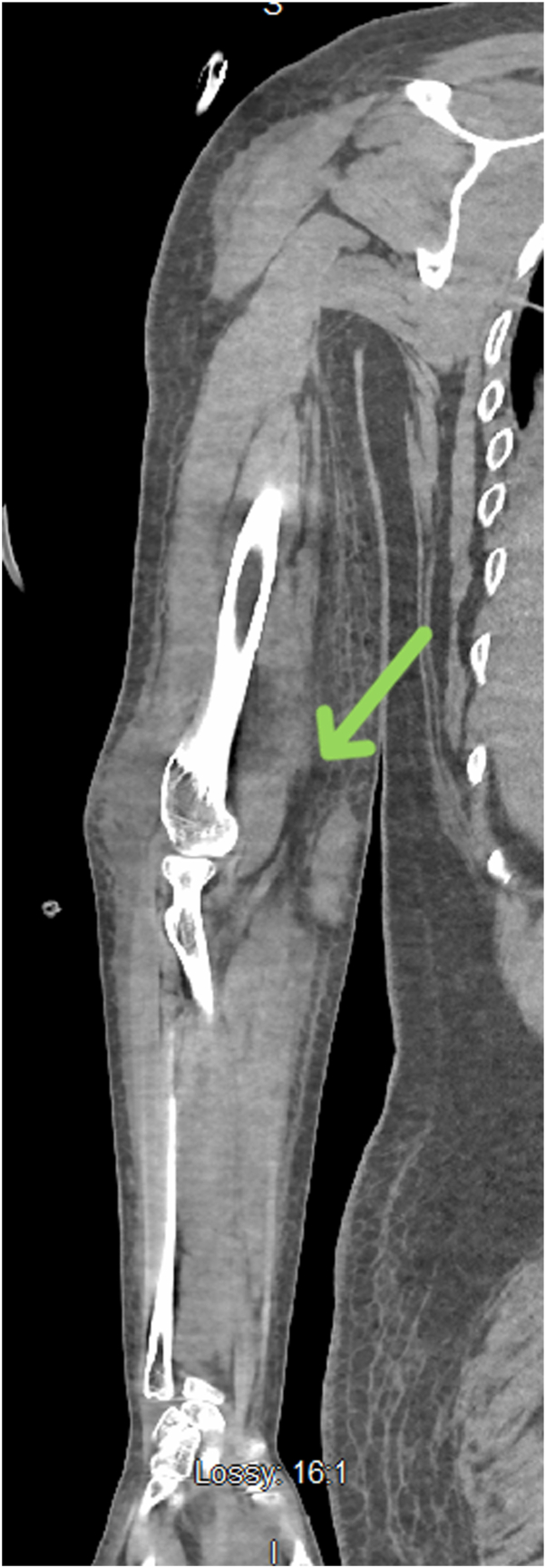
Diffuse infiltration of subcutaneous fat. A density fluid is seen in the subcutaneous fat (*arrow*), lateral and medial to the elbow. No soft tissue gas. Impression is right upper extremity cellulitis.

Subtle clinical picture, change in care team due to transfer to intensive care unit, and patient's nonverbal state due to intubation led to a delay in diagnosis. After 5 days, clinical staff noticed significant ecchymosis, bruising, and swelling, with the presence of a hematoma on the right anterior forearm. The right posterior forearm showed a small fluid collection, and the patient exhibited reduced mobility of the affected arm. Over time, a mixed serosanguineous, foul-smelling, and purulent discharge was noticed. The right upper extremity became tense, with 2+ pulses, bluish fingertips, and slow capillary refill (3-5 seconds). The patient had persistent leukocytosis. Due to concern of necrotizing fasciitis, a CT scan without contrast of the right arm was obtained, which revealed right upper extremity cellulitis, which could represent an abscess, hematoma, or fragment, but was negative for gas (Fig 2). Additionally, a Doppler study ruled out deep vein thrombosis. Clinical signs did not support compartment syndrome. Both blood and wound cultures showed no growth.

Initial suspicion of necrotizing fasciitis, patient was started on IV vancomycin, cefepime, and clindamycin. Later, General Surgery was consulted, and patient underwent extensive wound debridement, which required 3 different procedures. Eventually, patient received a right split-thickness skin graft, and he gradually regained full functionality of his right arm.

**Fig 3:**
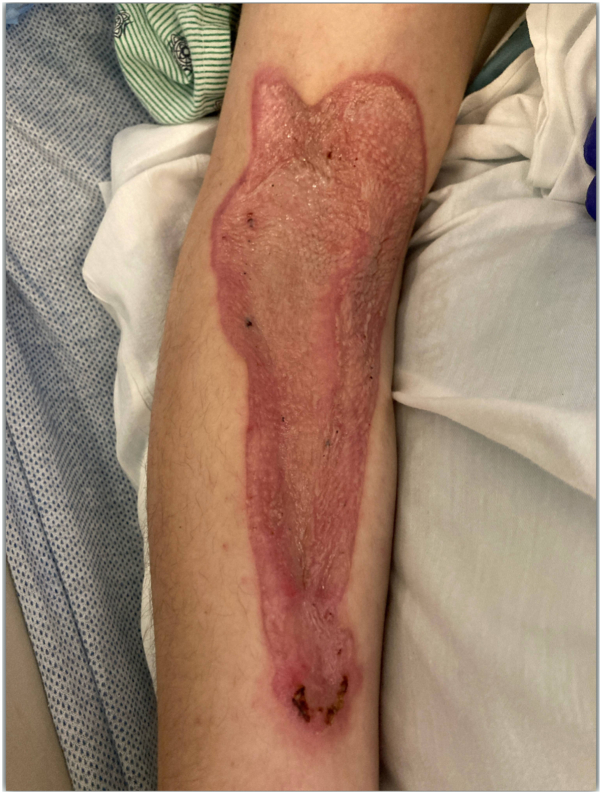
Healthy appearing graft tissue on 3-m follow-up.

The pathology of the wound tissue showed multiple dilated veins with congestion and organizing thrombi, as well as occasional veins with acute inflammation within the vessel walls, without fibrinoid necrosis. Extensive acute inflammation and necrosis were noted in the subcutaneous tissue of the debrided right arm. Necrotizing fasciitis was ruled out, as both blood and wound cultures were negative. The patient followed up with general surgery 3 months later with healthy graft tissue (Fig 3).

## Discussion

The complication of amiodarone-induced skin necrosis is irreversible tissue damage, which can sometimes lead to cosmetic disfigurement, scarring, or functional impairment. The extravasation site can initially appear erythematous and swollen, later progressing to ulceration and necrosis if left untreated. In severe cases, skin necrosis leads to extensive tissue loss that may require surgical intervention, such as debridement or skin grafting.^5^ As Jaworski et al ^6^ noted, necrosis can impair the function of the skin, most pronounced in areas like the hands, face, and other cosmetically relevant areas, presenting both physical and psychological challenges for patients. Additionally, delayed recognition and management may lead to complications such as infections of the affected tissue, complicating the recovery process.

Based on the case reports published on amiodarone extravasation, diagnosis is generally based on clinical signs such as swelling at the IV site, erythema, skin color changes and warmth, as well as patients' symptoms such as significant pain (usually >5 out of 10 on pain scale), decreased range of motion of affected limb and expanding induration. These clinical signs and symptoms are apparent in 6-24 hours after extravasation.^5^^,^^7^^,^^8^ In our case report, clinical signs did not appear until 5 days after extravasation, and the nonverbal status of the patient resulted in delayed presentation.

The clinical manifestations of amiodarone-induced skin necrosis highlight that management strategies, early recognition, and timely intervention play a vital role in addressing the condition. The first step is to stop the infusion of amiodarone to prevent further tissue damage and extravasation. As Epstein et al ^1^ suggest, when amiodarone extravasates into the surrounding tissues, appropriate measures must be taken to minimize the damage. Conservative management involving cold compresses is sufficient for less severe cases, helping to reduce inflammation and prevent further drug reabsorption. In more intensive cases, intradermal hyaluronidase has shown a promising role. Fox et al ^5^ demonstrated that hyaluronidase helps disperse the drug by breaking down extracellular matrix components, allowing the drug to diffuse away from the affected tissue site and reducing necrotic effects. In our case, physicians may have used intradermal hyaluronidase earlier despite the lack of obvious clinical signs, taking into account the unusual circumstances due to the nonverbal status of the patient.

In more severe cases, particularly those involving significant necrosis, surgical intervention would be the best possible approach. This response would include debridement of necrotic tissue, facilitating the healing process. In certain cases, skin grafts would be beneficial to cover extensive wounds, especially in cosmetically important areas. Additionally, Grove Al ^9^ discussed the role of early surgical intervention in preventing long-term consequences such as scarring or functional deficits. It also helps improve recovery time and quality of life for the individual.

## Conclusion

Amiodarone-induced skin necrosis, while rare, poses a significant challenge in clinical practice due to its potential for severe local and systemic complications. Early detection, appropriate medical and surgical interventions, and preventive strategies are key to managing this serious adverse effect. Through a multidisciplinary approach, including dermatologists, pharmacologists, and surgeons, the outcomes of patients can be optimized.

## Conflicts of interest

None disclosed.
